# Tobacco, Alcohol and Drug Use among Dental Undergraduates at One UK University in 2015

**DOI:** 10.3390/dj4010002

**Published:** 2016-01-25

**Authors:** James Puryer, Rebecca Wignall

**Affiliations:** School of Oral and Dental Sciences, Bristol Dental Hospital, Lower Maudlin Street, Bristol BS1 2LY, UK; rebecca.wignall@bristol.ac.uk

**Keywords:** undergraduate, tobacco, alcohol, smoking, drugs

## Abstract

The aim of this study was determine the prevalence of tobacco, alcohol and illicit substance use among dental undergraduates at one UK university in 2015. A cross-sectional survey of all 344 dental undergraduates using an anonymous self-report questionnaire was carried out. The response rate was 77%, of which 29% were male and 71% female. Tobacco smoking was reported by 23.6% of males and 12.2% of females, with only 1.6% of females reporting to smoke ≥10 cigarettes per day. Alcohol consumption was reported by 85.5% of males and 84% of females, and reported levels of alcohol consumption increased since becoming undergraduates. Binge drinking was reported by 35.3% of males and 41% of female students. Only 2.6% of males and 0.5% of females reported to be current regular users of cannabis. The vast majority of respondents claimed to have never used any illicit substance. The only other reported regularly used substances by males was Ecstasy (1.3%) and by females were LSD (0.5%), Ecstasy (1.5%), Cocaine (0.5%), Inhalants (0.5%) and Ketamine (0.5%). These results are encouraging. Fewer students reported smoking than in the general population, levels of binge drinking were considerably lower than previously reported figures, as were the numbers of regular users of cannabis and other illicit substances.

## 1. Introduction

Alcohol and drug misuse among UK undergraduates has been a recognised problem for many years [1,2,3], and previous studies of dental undergraduates [4,5,6,7] suggest that they are no exception. This behaviour does not appear to be restricted to the UK, as research carried out in other parts of the world has shown similar results [8,9,10]. It has been previously reported [5] that an increasing number of male (69.5%) and female (66.1%) dental undergraduates undertook binge drinking, despite the overall consumption of alcohol falling. The problem of binge drinking is of particular concern due both to the associated antisocial behaviour and health risks [11,12] and also its prevalence. It is expected that alcohol-related harm will cost society £21 billion per year in healthcare, lost productivity costs, crime and anti-social behaviour [13]. The same study reported that 27% of male and 13.5% of female dental undergraduates smoked tobacco and that this number had decreased from their earlier survey. The study also concluded that whilst the use of some illicit drugs (cannabis and amphetamines) were less common than previously, there was an increase in the use of amyl nitrate, especially amongst females.

The effects of excessive alcohol intake and the use of illicit recreational drugs have potential serious consequences for the careers of dentists. A recent publication by the General Dental Council (GDC) [14] does not specifically mention the use of alcohol and drugs, although standard 9.1 states *“Ensure that your conduct, both at work and in your personal life, justifies patients’ trust in you and the public’s trust in the dental profession”.* In addition, another recent GDC document gives guidance on how convictions relating to drugs and alcohol amongst dental professionals are assessed in related to Fitness to Practise cases [15]. Ultimately, a charge of serious professional misconduct can arise relating to an allegation of ‘drunkenness or the misuse of drugs’, as detailed in an earlier GDC guidance document [16].

Current government guidelines [17] recommend that men should not regularly drink more than 3–4 units of alcohol per day, and that women should not regularly drink more than 2–3 units of alcohol per day. “Regularly” means drinking every day or most days of the week. These guidelines also state that drinking in excess of this can damage a person’s health, and that alcohol is one of the biggest behavioural risks for disease and death (as well as smoking, obesity and lack of physical activity). In 2010 to 2011 there were 1.2 million alcohol-related hospital admissions and around 15,000 deaths caused by alcohol [17].

The Office for National Statistics (ONS) has recently produced a series of recent publications looking at the prevalence of alcohol, smoking and drug misuse amongst the general population in England: [18,19,20]

Within the general population, 20% of adults aged 16 and over were smokers in 2012, a rate that has remained largely unchanged in recent years, compared to 26% a decade earlier in 2002 [18]. The prevalence of smoking among men (22%) was slightly higher than for women (19%). When looking specifically at young people aged 20–24, the rate has fallen from 35% to 29%, and it is this age group that still has the highest prevalence of smoking within the general population. [18]

With regards alcohol consumption within the general population, there has been a gradual decline such that between 2005 and 2012 the proportion of men who had drank alcohol in the week before being interviewed fell from 72% to 64%, and the proportion of women fell from 57% to 52% [19]. Young people (those aged 16–24) were more likely to have binged on alcohol at least once during the week, with a similar proportion for men (26%) and women (28%) [19].

With regards drug misuse within the general population, 9% of all adults aged 16–59 had taken an illicit drug within the last year [20]. However, this proportion has more than doubled (19%) when looking at the age subgroup of 16–24 year-olds. This has risen from 8% of 16–59 year olds and 16% of 16–24 year-olds when compared to the previous year. The use of cocaine, ecstasy, LSD, ketamine and New Psychoactive Substances (NPS) (also known as ‘Legal Highs’) had particularly increased. In fact, a recent study [21] found that the UK accounts for 23% of world consumption for NPS. Young adults were more likely to be frequent drug users than older people and 6.6% of young adults aged 16–24 were classed as frequent drug users [20].

## 2. Aims

The aim of this study was to determine the prevalence of tobacco, alcohol and recreational drug use among current dental undergraduates at one UK University in 2015.

## 3. Methods and Materials

Full ethical approval from the Faculty of Medicine and Dentistry Committee for Ethics was obtained prior to the study—FREC No. 16861.

A cross-sectional survey of all dental undergraduates (n = 344) studying in years 1 to 5 at one UK university in 2015 was carried out. The questionnaire was based upon the one used in the 2008 survey by Underwood *et al.* [5] and only modified by the addition of ‘Ketamine’ and ‘Legal Highs’ to the option list of recreational drugs. As the questionnaire had been previously used successfully, no pilot study was undertaken. The questionnaire, consisting of four sides of A4 paper (copy available from corresponding author), was designed to investigate the frequency of tobacco smoking, alcohol and recreational drug use both whilst an undergraduate and before becoming an undergraduate. A closed-style of questioning was used, with all of the questions being ‘tick-box’ responses to aid speed of completion and anonymity, as well making quantitative statistical analysis possible.

The questionnaire was distributed to undergraduate students by year group at the end of one of their compulsory lectures during the period 10 March–2 June, 2015. The students were e-mailed in their year groups one week prior to these lectures to explain the nature of the survey, reassure students that it was anonymous. Participation was non-compulsory. Seating in the lecture theatre allowed students to be adequately separated from their peers in order to maintain confidentiality of responses. Completed questionnaires were deposited in a box as students left the lecture theatre.

Anonymity of participants was essential, and thus no name, age or ethnic origin was requested. For this reason, no follow-up e-mail inviting those individual students who were not present at the lecture to complete the questionnaire was sent as it would have been difficult to maintain anonymity of responses.

## 4. Results

Questionnaires were completed by n = 264 undergraduates, out of a possible n = 344. All of the questionnaires were completed in full and were included in the study, which gave a usable response rate of 77%. 29% of respondents were male and 71% female. The demographics of the respondents are shown in Table 1.

**Table 1:** The demographics of respondents (n = 264)

Male	76 (29%)
Female	188 (71%)
Year-1	45 (17%)
Year-2	68 (26%)
Year-3	56 (21%)
Year-4	33 (13%)
Year-5	62 (23%)

**Tobacco smoking:** The prevalence of tobacco smoking by undergraduates (defined as those who currently smoke whilst drinking alcohol, smoking cannabis, or those who regularly smoke ≥10 cigarettes per day) was n = 18/76 (23.6%) for males and n = 23/188 (12.2%) for females. Current tobacco smoking of ≥10 cigarettes per day was reported by n = 0/76 (0%) males and n = 3/188 (1.6%) of females. This was a lower percentage than the 2008 study [5] where it was found that 4.3% of males and 4.3% of females were regular tobacco smokers. N = 33/76 (43.4%) of males and n = 99/188 (52.6%) of females claimed never to have smoked tobacco (Table 2).

**Table 2:** Current smoking habits of respondents (n = 264)

Frequency of tobacco use	Male (n = 76)	Female (n = 188)
I have never smoked tobacco	33	99
I have tried tobacco, but did not continue	24	62
I smoke tobacco only whilst drinking alcohol	17	17
I smoke tobacco only with cannabis	1	3
I have been a regular (10+ cigarettes per day) smoker, but not in my current year	1	2
I have been a regular (10+ cigarettes per day) tobacco smoker in my current year, but not now	0	2
I currently smoke tobacco on a regular (10+ cigarettes per day) basis	0	3

There was an overall reduction in tobacco smoking reported since becoming an undergraduate as n = 3/76 (3.9%) of males and n = 4/188 (2.1 %) of females reported to have previously smoked ≥10 cigarettes per day (Table 3).

**Table 3:** Previous smoking habits of respondents (n = 264).

Frequency of tobacco use	Male (n = 76)	Female (n = 188)
I have never smoked tobacco	43	117
I have tried tobacco, but did not continue	19	51
I smoked tobacco only whilst drinking alcohol	10	14
I smoked tobacco only with cannabis	1	2
I was regular (10+ cigarettes per day) smoker	3	4

**Alcohol consumption:** The prevalence of alcohol consumption by undergraduates was n = 65/76 (85.5%) of males and n = 158/188 (84%) of females. Thus 14.5% of males and 16% of females claimed never to consume alcohol. The levels of alcohol consumption reported have increased since becoming undergraduates as n = 14/76 (18.4%) of males and n = 43/188 (22.8%) of females claimed never to drink alcohol prior to starting university. This is reflected in answer to the question on how students perceived their weekly consumption of alcohol compared to before becoming an undergraduate, as n = 41/76 (53.9%) of males and n = 87/188 (46.2%) of females reported that their level of alcohol consumption was less before starting their studies. Only n = 11/76 (14.5%) of males and n = 18/188 (9.6%) of females reported that they consumed more alcohol on a weekly basis before starting their studies.

The current drinking patterns of undergraduate students by gender is shown in Table 4. (NB the sum of percentages do not equal 100% due to ‘rounding’ of small sample sizes). Of those students that consumed alcohol the previous week, 47.6% of males and 66.4% of female only drank 0–7 units of alcohol, whilst only 1.8% of female students reported drinking greater than 42 units. Binge drinking (≥10 units for males and ≥7 units for females in an average drinking session) was reported by 35.3% of males and 41% of female students.

**Table 4:** Current drinking habits of undergraduates (n = 223)

Units	Male (n = 65)	Female (n = 158)
**Units of alcohol drunk in the last week (%)**		
0–7	47.6	66.4
8–14	21.5	15.1
15–21	9.2	7.5
22–28	13.8	2.5
29–35	6.1	4.4
36–42	1.5	1.8
43–49	0	0.6
50+	0	1.2
**Units of alcohol drunk in an average drinking session (%)**		
0–3	10.7	21.5
4–6	30.7	37.3
7–9	23	26.5
10+	35.3	14.5

**Cannabis:** The current use of cannabis by gender of undergraduate students is shown in Table 5. n = 48/76 (63.2%) of males and n = 121/188 (64.4%) of females reported never to have used cannabis, whilst n = 2/76 (2.6%) of males and n = 1/188 (0.5%) of females reported to be current regular users. N = 0/76 (0%) of males and n = 3/188 (1.6%) of females reported to be regular users before starting their studies.

**Table 5:** Current undergraduate use of cannabis (n = 264)

Frequency of cannabis use	Male (n = 76)	Female (n = 188)
Never used	48	121
Used once or twice	13	33
Used more than twice	12	28
Previous regular user, but not in current year of study	1	4
Previous regular user, but not now	0	1
Current regular user	2	1

**Other illicit drugs:** The current use of other illicit substances by gender of undergraduate students is shown in Table 6. The vast majority of both male and female respondents claimed to have never used any illicit substance. The only reported regularly used substances by males was Ecstasy (1.3%) and by females were LSD (0.5%), Ecstasy (1.6%), Cocaine (0.5%), Inhalants (0.5%) and Ketamine (0.5%).

**Table 6:** Current undergraduate use of other illicit drugs (n = 264)

Illicit drug	Male (n = 76)						Female (n = 188)						
	Never Used	Used once or twice	Used more than twice	Previous regular user but not in current year	Previous regular user but not now	Current regular user	Never Used	Used once or twice	Used more than twice	Previous regular user but not in current year	Previous regular user but not now	Current regular user
Amphetamine	73	2	1	0	0	0	179	7	2	0	0	0
LSD	75	1	0	0	0	0	182	3	1	0	1	1
Ecstasy	64	7	2	2	0	1	156	14	13	2	0	3
Cocaine	70	5	1	0	0	0	178	4	3	1	1	1
Amyl Nitrate	73	3	0	0	0	0	181	6	0	0	1	0
Inhalants	75	1	0	0	0	0	187	0	0	0	0	1
Magic Mushrooms	72	3	1	0	0	0	179	7	2	0	0	0
Steroids	75	1	0	0	0	0	188	0	0	0	0	0
Ketamine	73	3	0	0	0	0	182	4	1	0	0	1
Legal High	69	1	5	1	0	0	183	2	2	1	0	0

**Associations:** Of the n = 41/264 (15.5%) students who claimed not to drink alcohol, only n = 1 (2.4%) has ever tried any illicit substance, and n = 41/41 (100%) students do not smoke. Only n = 13/132 (9.8%) of non-smokers have ever tried any illicit substance.

## 5. Discussion

This paper reports on tobacco, alcohol and recreational drug use among dental undergraduates at one UK University before admission and during their studies in 2015. The response rate of 77% compares favourably with previous similar studies carried out in 1998 (usable response rate of 76%) and in 2008 (usable response rate of 67%) [5]. The ‘gold standard’ of a 100% response rate was not achieved, and we can only speculate as to the reasons why. It is possible that non-responders were substance users that did not want to divulge their habits, or simply the fact that they could not recall their habits [19]. It is also possible that those students who frequently smoked or misused substances may not have wanted to complete the survey as it might have challenged their denial of any problem. Due to the strict anonymity of the survey, students should not have felt under any pressure to be dishonest in their response and thus we assume that all responses were honest.

The prevalence of tobacco smoking (23.6 % males and 12.2% females) is less than both that of the figures reported in the 2008 study [5] and also within the general population, where the prevalence of smoking in the 20–24 year old range is 29% [18]. The prevalence of smoking is twice as common in males as it is female, and is it encouraging that the overall trend is of a reduction in smoking levels. There was an overall reduction in tobacco smoking since becoming an undergraduate also. The reasons for this may be many, and may include current UK legislation relating to smoking (cigarette advertising banned on billboards, in the press and media (2003), restrictions on advertising at the point of sale (2004), smoking banned in enclosed areas (2006) and the illegality of selling tobacco products to minor under the age of 18 years (2007)), as well as the increasing stigma that is becoming associated with tobacco smoking in recent years. Coupled with this, dental students are expected to be able to give appropriate advice to patients with regards smoking cessation advice [22] and it is not unreasonable to expect that some students take heed of this advice themselves, along with their growing maturity and professional standards as they progress through the course. This downward trend in smoking prevalence is mirrored in vocational dental practitioners [23].

Current smokers within this cohort of undergraduates are few in number, with not a single male and only 1.6% of females reporting to smoke ≥10 cigarettes per day, and again, this is less than the 2008 study [5] where 4.3% of both male and female students reported to smoke tobacco regularly. Whilst it is not possible to predict future tobacco smoking habits by undergraduates, this apparent trend is extremely encouraging.

A significant number of undergraduates drink alcohol (85.5% of males and 84% of females), which is higher than the figures reported within the general population [19]. This prevalence has increased since becoming undergraduates, and 53.9% of males and 46.2% of females admitted that they drunk less alcohol before starting their studies. These results are unsurprising as undergraduate students are now at a legal age to purchase alcohol, and in addition, the culture of being a university student is likely to have a role [24]. These figures support the study carried out in 2008 [5]. However, despite this prevalence, the majority of students who drink alcohol do so in moderation, with 47.6% of males and 66.4% of females only consuming 0–7 units of alcohol within the previous week. Only 7.6% of males and 9.3% of females drank over the current government guidelines [17] of 28 and 21 (respectively) units of alcohol in the previous week.

In order to allow comparisons with previous studies, the current study used the same definition of binge drinking (≥10 units for males and ≥7 units for females in an average drinking session) as the 2008 study [5]. The current reported levels of binge drinking (35.3% of males and 41% of female students) were considerably lower than the figures reported in the 2008 study [5] where 69.5% of males and 66.1% of females reported to binge drink. This trend of decreasing levels is also reflected within the general population and is encouraging. This trend may be attributable to government health campaigns and policies [25] to raise the number of units of alcohol that drinks contain and subsequent increased student awareness, or it may be simply the diversity of the populations studied. Students may also be restricting the amount of alcohol that they consume within the week due to clinical commitments and subsequently binge at the weekends. The reasons for this trend are unclear, and further research is needed in this area. It would be difficult to overlook the enjoyment associated with drinking alcohol, but there reaches a point for every individual where the risks outweigh the benefits. Despite formal guidance being given to undergraduates as part of their dental education, there is still evidence of heavy drinking by a number of undergraduates and further education or intervention is needed to avoid any unwanted consequences.

The prevalence of cannabis smoking reported within this study is very low, and this is particularly encouraging as the trend within the general population is that of an increased use [20]. Whilst the numbers of students who reported never to have tried cannabis are similar to the 2008 study [5] (63.2% male, 64.4% female *vs.* 62.0% male, 67.7% female), only 2.6% of male and 0.5% of female students report to currently use cannabis. These percentages are lower than those of the 2008 study [5] where 5.4% and 0.6% of students reported to be regular users. The reason for this reduction (especially for males) is again unclear. However, one possible explanation is that in 2009 the government reclassified cannabis from Class C to Class B [26], and students are mindful of the greater risks involved if caught in the possession of cannabis, with possible criminal convictions and wider ramifications for future employment or even registration.

The vast majority of students (96.0% male and 95.2% female) claimed never to have used any illicit substance, and the only regularly used substances reported by males was Ecstasy (1.3%) and by females were Ecstasy (1.6%), LSD (0.5%), Cocaine (0.5%), Inhalants (0.5%) and Ketamine (0.5%). These very encouraging figures support the trend of reduced use found in the 2008 survey [5] compared to the 1998 [4] survey. Again, the reasons for this are unclear and further research could be carried out in this area.

It was difficult to find any strong associations between alcohol, smoking, and illicit drug use due to the very few numbers who reported using these substances. However, it was not surprising that of the 15.5% of all students who did not drink alcohol, none of them smoked and only 2.4% had tried any form of illicit substance.

The current study does have some limitations. It would be incorrect to directly compare the results of this study with reports of substance use within the general population due to lack of uniformity of methodologies and definitions of usage. Only trends can be made between the results of this study and those in the general population. Similarly, whilst, references to the survey carried out in 2008 can be made due to both having very similar methodologies and use of an almost identical questionnaire (with only a single question having being modified), no direct comparisons can be made as this survey was carried out at another institution. In addition, there is a notable difference in gender balance between earlier studies and the current study where the proportion of female respondents has now risen to 71%. This may certainly have had an effect on the altered patterns of alcohol and substance use. Furthermore, whilst the current responses give a ‘snapshot’ of substance use, as does the 2008 survey, any trends that have occurred over the 7 year period may not be ‘linear’. Thus, it is difficult to make future predictions of substance use amongst dental undergraduates.

In terms of study generalisability, the dental undergraduates surveyed at a single UK university may not be representative of all UK dental undergraduates in the substances they use or in the patterns of their use. Other UK dental schools may have differing numbers of mature students, or students from ethnic minorities. Higher abstention rates from alcohol consumption, and less tobacco and cannabis use by ethnic minority students compared to white students have previously been reported [1,27,28], and tobacco and alcohol use within the general population also varies with ethnic origin. Whilst it would have been useful to have data concerning undergraduate age and ethnicity, these questions were not asked so as to maintain anonymity and not risk identifying individual respondents.

No questions were asked as to *why* a particular substance was used, or *how* an individual substance was used. Differing methods of preparation and subsequent use of a drug may have different associated risks. Similarly, no questions were asked as to *when* various substances were used in relation to term-time *vs.* vacations or weekends *vs.* midweek. However, there is no evidence that tobacco use, alcohol consumption and/or the use of illicit substances reported by dental undergraduates has had any detrimental effect in either the care that they provide to their patients or their academic performance.

Recall bias may be a problem when answering questions about substance use before becoming a dental undergraduate.

Despite these limitations, the methodology of the study and the usable response rate of 77% justifies in making its findings relevant to the current evidence base, and provides a more contemporary benchmark of a current overview at a single university from which further studies could be conducted. The successful use of this questionnaire could be repeated at all UK Dental Schools (if funding and approval could be obtained), and would give a comprehensive picture of substance use amongst UK dental undergraduates. The study could also be expanded to study dental undergraduates in other countries. It would support the suggestion [5] from the 2008 study of trialling different harm reduction and primary prevention programs to allow comparison as to the most effective. These could then be introduced throughout the UK Dental Schools in order to further reduce the positive trends reported in this study.

## 6. Conclusion 

The results from this survey are very positive, with this current cohort of students at one UK University showing reduced levels of drinking, smoking and illicit substance use than in four previous studies [4,5,6,7]. Further research could be carried out to investigate use within other Dental Schools, and also investigate further the reasons behind these encouraging trends.
